# Supplementary material for: Ca2+-Triggered Coelenterazine-Binding Protein Renilla: Expected and Unexpected Features
Source: Int J Mol Sci. 2023 Jan 21;24(3):2144. doi: 10.3390/ijms24032144 (PMC9917264; doi:10.3390/ijms24032144)
Supplement: Supplementary file 1 [file ijms-24-02144-s001.zip › ijms-2138068-supplementary.pdf]

Expected and Unexpected Features of Ca^{2+}-triggered Coelenterazine-Binding Protein Renilla

Alexander N. Kudryavtsev, Vasilisa V. Krasitskaya, Maxim K. Efremov, Sayana V. Zangeeva, Anastasia V. Rogova, Felix N. Tomilin, Ludmila A. Frank

Supplementary materials.

1. Purity of the recombinant NanoLuc apoCBP, CBP and FMZ-apoCBP, used in the study

Codon-optimized gene of NanoLuc luciferase was expressed in *E. coli* cells. According to SDS-PAGE data, the protein was almost evenly distributed between the cytoplasm and the pellet of inclusion bodies and cell debris (Figure S1, left). Luciferase of high purity was obtained from both fractions but its bioluminescence activity from inclusion bodies was 50-fold lower than that from cytoplasm (data not shown). So, for the following experiments NanoLuc obtained from cytoplasm was used (Figure S1, on the left). The yield of high purity NanoLuc was 150-200 mg per 1 liter of LB media.

Purity of the recombinant apoCBP, CBP and FMZ-apoCBP preparations is shown in Figure S1, right.

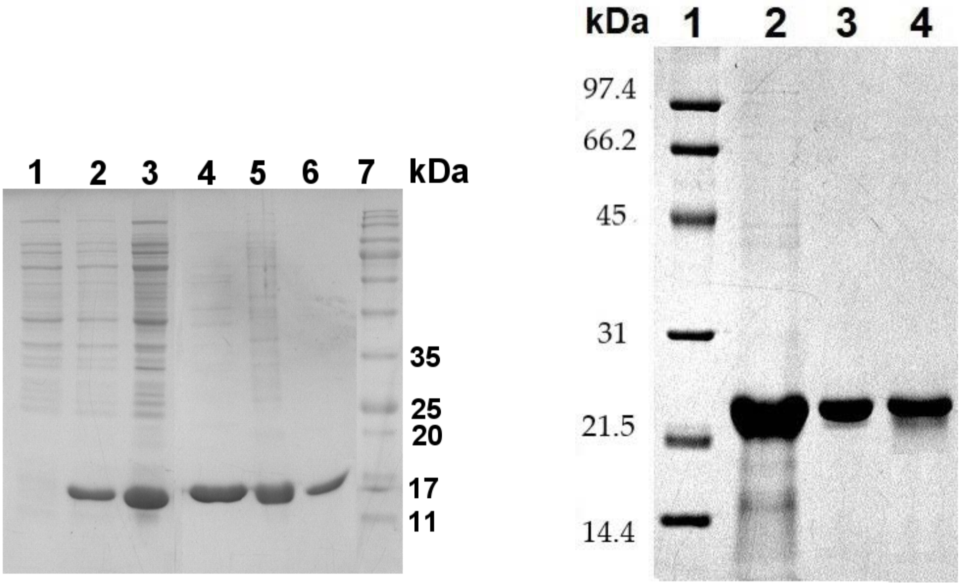

Figure S1. On the left: 12.5% SDS-PAGE analysis of the NanoLuc during purification. Lanes: 1,2 – whole-cell lysates before and after IPTG induction, respectively; 3 cytoplasm fraction; 4 – NanoLuc after purification from cytoplasm; 5 – 6 M urea extract of inclusion bodies; 6 – NanoLuc after purification from inclusion bodies; 7 – standard proteins. **On the right**: 12.5% SDS-PAGE analysis of the proteins, used in the experiments: apoCBP (lane 2); CBP (lane 3); FMZ-apoCBP (lane 4). Standard proteins: lane 1. Molecular masses of standard proteins are shown with numbers.

2. Chromophore (establishment of a chromophore group)

The molecular orbitals of CTZ and FMZ were examined to identify the group of atoms that are key role to electronic excitation. (Figure S2 a, b). The shape of the boundary molecular orbitals suggests that the chromophore group is localized on heterocycle R (R') and substituent R_1 (R'_1), both for coelenterazine and furimazine. As shown in the figure of boundary orbitals for CTZ, HOMOs are mainly localized near the

pyrazine ring and R_1 (R'_1). The electron density LUMO orbitals are already at R (R') for CTZ and FMZ, respectively. It can be concluded that the chromophore of this molecule is the R and R_1 groups and is the same for these substrates. So, replacement of phenolic rings (R_1, R_3) by furan (R'_3) and benzene (R'_1) does not lead to changes in the chromophore group and spectral properties.

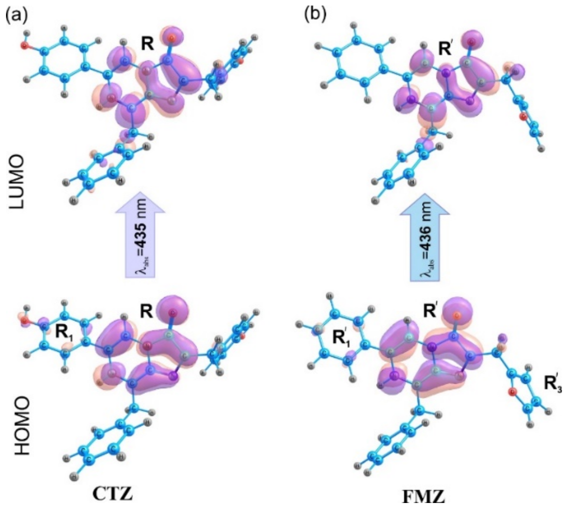

Figure S2. The molecular orbitals of CTZ and FMZ involved in absorption transition $S_0 \rightarrow S_1$. (a) – CTZ, (b) – FMZ. λ_{abs} – maximum in the absorption spectra in nm. Calculations in TD/SMD methanol/B3LYP/cc-pVDZ level of the theory.

3. Bioluminescence spectra of NanoLuc luciferase with the different substrates

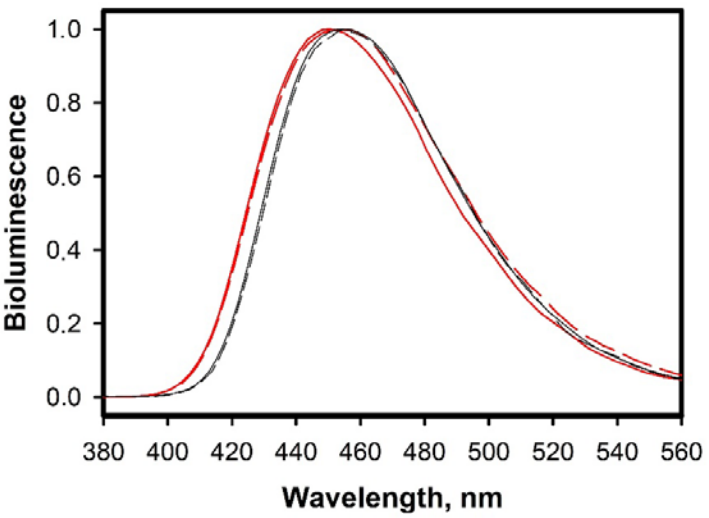

Figure S3. Normalized bioluminescence spectra of NanoLuc luciferase with furimazine (red line), FMZ-apoCBP (red dotted line), coelenterazine (black line) and CBP (black dotted line).

4. NanoLuc bioluminescence with the different substrates

Table S1. Apparent kinetic parameters of the NanoLuc bioluminescent reactions

Substrate	K_M, μM	V_{max}, 10^4 r.l.u. s^{-1}	k_{cat} 10^4 s^{-1}	k_{cat}/K_M $10^5 \mu M^{-1}$ s^{-1}
CTZ	0.22	4.6	4.6	2.1
CBP	0.47	7.8	7.8	1.8
FMZ	0.60	26.1	26.1	4.5
FMZ-apoCBP	0.45	8.6	8.6	1.9

K_M - Michaelis constant; **V_{max}** –maximal velocity of the reaction; **k_{cat}** – turnover number
